# Biostimulant enhances growth and corm production of saffron (*Crocus sativus* L.) in non-traditional areas of North western Himalayas

**DOI:** 10.3389/fpls.2023.1097682

**Published:** 2023-02-15

**Authors:** Neha Chaudhary, Deepak Kothari, Swati Walia, Arup Ghosh, Pradipkumar Vaghela, Rakesh Kumar

**Affiliations:** ^1^ Agrotechnology Division, Council of Scientific and Industrial Research (CSIR)-Institute of Himalayan Bioresource Technology, Palampur, India; ^2^ Academy of Scientific and Innovative Research (AcSIR), Ghaziabad, India; ^3^ CSIR- Central Salt and Marine Research Institute, Bhavnagar, Gujarat, India

**Keywords:** *Crocus sativus*, seaweed extract, growth, biochemical, nutrients, corm production

## Abstract

The usage of seaweed extracts in cropping systems is gaining attention nowadays due to their distinct bioactive properties. This study aims to assess how saffron (*Crocus sativus* L.) corm production was affected by seaweed extract through different application modes. The study was conducted at the CSIR-Institute of Himalayan Bioresource Technology, Palampur, HP, India, during the autumn-winter agricultural cycle. Five treatments using a combination of Kappaphycus and Sargassum seaweed extracts were replicated five times in a randomized block design. Treatments that were examined include T1: Control, T2: Corm dipping @ 5% seaweed extract, T3: Foliar spray @ 5% seaweed extract, T4: Drenching @ 5% seaweed extract, and T5: Corm dipping + foliar spray @ 5% seaweed extract. Seaweed extract, when applied to saffron plants (T5: Corm dipping + foliar spray @ 5% seaweed extract) resulted in significantly higher growth parameters along with the higher dry weight of stem, leaves, corms, and total roots per corm. Corm production, *viz*., the number of daughter corms and corm weight per m^2^ was significantly affected by seaweed extract application, with the maximum value recorded with treatment T5. Biochemical parameters chlorophyll, carotenoids, and photosynthetic rate were higher in T5, while nutrient concentration was lowest in this treatment. Seaweed extracts improved corm production, making it a feasible alternative to limiting the application of conventional fertilizers, attenuating the effects on the environment, and enhancing corm number and weight.

## Graphical Abstract

Application of seaweed extract increased the corm production of saffron. 
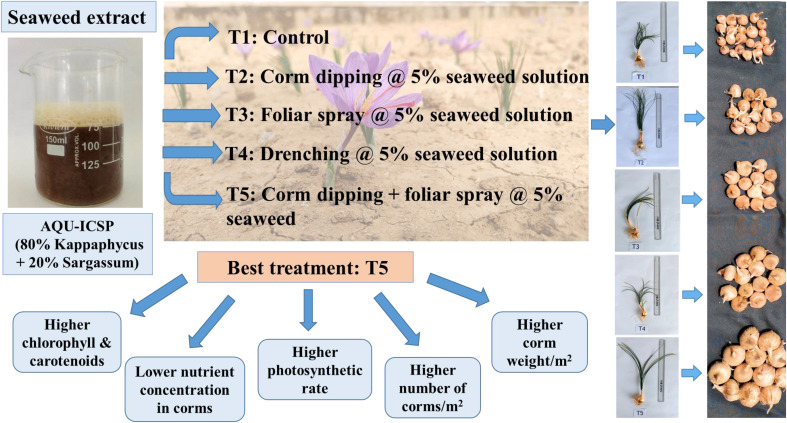

## 1 Highlights

- Application of 5% seaweed extracts by corm dipping + foliar spray enhanced saffron corm yield.
- Increase in number of daughter corms as well as weight of corms per m^2^ led to yield increase.
- Nutrients in corms were lowest because of efficient uptake increasing corm production
- Seaweed extracts enhanced chlorophyll and photosynthetic rate.
- Seaweed extracts lower carbon footprints and can lower global warming leading to cleaner production.

## 2 Introduction

Saffron (*Crocus sativus* L.) is one of the costliest and oldest cash crops (family Iridaceae) among the aromatic and medicinal plants (Mohtashami et al., 2021) and holds great value in the global market, with an average price of 1500-2200 €/kg (Mykhailenko et al., 2020), hence also known as the ‘red gold’ (Shahi et al., 2016). It derives its name from the Persian name ‘Zaafaran’ which means yellow flowers (Ramadan et al., 2010). Saffron is a native of the Mediterranean region and is grown throughout the world between Spain to Kashmir (longitudinally) and Persia to England (latitudinally) (Khan et al., 2011). In the Indian Union Territory of Jammu and Kashmir, saffron is grown in the Pulwama, Budgam, Srinagar, Doda, and Kishtwar districts (Kumar and Sharma, 2017). Due to the fact that saffron is a sterile triploid plant, it is naturally multiplied *via* daughter corms (Bayat et al., 2016; Mir Shafat et al., 2021). The Crocus flower’s dried red stigmas are its most valuable component. Each fresh flower weighs 300–500 mg approximately with fresh and dry stigmas weight around 25–47 mg and 6–7 mg, respectively, therefore around 160,000– 110,000 saffron flowers are needed to get 1 kg of spice (Mansotra and Vakhlu, 2022). Saffron is grown in a variety of settings with varying pedo-climatic conditions, and its estimated world annual production is 418 t (Cardone et al., 2020). Iran (108,000 ha), Afghanistan (7,557 ha), India (3,674 ha), Greece (1000 ha), Morocco (850 ha), Spain (150 ha), Italy (70 ha), and France (37 ha) are the main producers of saffron (Ganaie and Singh, 2019; Koocheki et al., 2019; Cardone et al., 2020; Mohammadi and Reed, 2020). With 80% of the world’s output, Iran is recognized as the world’s top producer (Menia et al., 2018; Kothari et al., 2021a; Kothari et al., 2021b; Kumar et al., 2022).

Despite its widespread appeal, which allowed all of the countries that produce saffron to boost production during the past 30 to 40 years, saffron production has decreased in all of the countries except for Iran in recent years (Ganaie and Singh, 2019; Cardone et al., 2020; Shahnoushi et al., 2020). To avert a severe reduction in saffron production globally, immediate action is needed. Therefore, it is crucial that saffron cultivation and processing increase profitability. The most expensive input in the growth of saffron is corm. About 5 lakh/ha of corms (10 g per corm) were needed if spaced 20 x 10 cm apart, which comes out to be 3015.82 US $ per hectare (INR 250000/ha) at 603.16 US $ per ton (INR 50000/t) market rate (Alam, 2006), however, this rate is increasing with an upward trend and reported around Rs 250-300 per kg corms in 2022 (personal communication). Ten to twelve year-long planting cycles used by Kashmiri saffron growers significantly reduce the likelihood of corm availability in India (Nehvi et al., 2010). Any effort to apprise saffron growing will consequently need an efficient mass corm manufacturing process because saffron reproduces vegetatively through corms. Additionally, saffron has attested to the significance of corm size in raising saffron yield (Alam, 2006). Therefore, it is understood that understanding saffron corm behavior for the production of daughter corms is important.

A great deal of research has been conducted globally to enhance saffron output as well as lower production costs (Maggio et al., 2006; Mzabri et al., 2021). One way to increase output, regulate plant flowering, reduce negative environmental impact and induce sustainability is by using plant growth regulators or biostimulants (Rathore et al., 2009; Sharma et al., 2016). Biostimulants are ‘materials other than fertilizers and pesticides’ that stimulate nutritional processes independent of the crop’s nutrient content with the specific aim of enhancing nutrient use efficiency, resilience to abiotic stress, quality traits, or availability of confined nutrients in the soil or rhizosphere (Rouphael and Colla, 2020). Among these biostimulants, the use of seaweed extract has gradually replaced conventional synthetic fertilizers as a source of natural organic fertilizer (Begum et al., 2018). One-third of the earth’s surface is covered with water consisting of natural and abundant sources of seaweed. Regardless of the amount of nutrients present, seaweed extracts are frequently employed as plant biostimulants, which are defined as “any substance or microbe administered to plants with the purpose of enhancing nutrition efficiency, abiotic stress tolerance, and/or crop quality attributes” (Du Jardin, 2015; Ali et al., 2021a). Seaweed extracts are expected to account for more than 33% of the global market for biostimulants and reach a value of 894 million Euros by 2022 (Eef et al., 2018; El Boukhari et al., 2020). A wide range of benefits, including improved yields and quality, have been reported with the use of marine bioactive substances derived from marine algae (seaweed extracts) in agricultural and horticultural crops (Battacharyya et al., 2015; Ali et al., 2021a). Variety of macronutrients, micronutrients, and organic substances such as sterols, amino acids, growth hormones, and vitamins are present in seaweed extract (Blunden, 1991; Sivasankari et al., 2006; Khan et al., 2009; Mondal et al., 2015).

Seaweed extract based biostimulants also contain quaternary ammonium compounds (Trivedi et al., 2018) and several other metabolites (Vaghela et al., 2022) which have been found to be associated with several plant physiological processes including that of tolerance to drought stress. Seaweed extracts have been reported to cause alterations in the physiological/biochemical processes involved in nutrient intake and plant development such as early seed germination and establishment (Calvo et al., 2014), accelerated root growth, increased leaf chlorophyll (Jannin et al., 2013; Kumar et al., 2020a), better production and greater tolerance to environmental stress (Yao et al., 2020). Enhanced enzymatic and non-enzymatic antioxidants and concomitant reduction of reactive oxygen species within the plant tissues were found to be responsible for such drought tolerance upon the application of seaweed extracts (Deolu-Ajayi et al., 2022). Seaweed extracts also have a beneficial impact on the biological, biochemical, chemical, and physical characteristics of the soil (Yao et al., 2020). Application of seaweed biostimulant derived from *Kappaphycus alvarezii* showed favorable effects on soil enzymatic activity and soil bacterial community under drought conditions (Trivedi et al., 2021a). Seaweed extracts can create colloids by combining soil metal ions and protecting the soil aggregate structure (Halpern et al., 2015). Seaweed extracts have grown in popularity as a result of their potential application in organic and sustainable agriculture as a way to refrain from overusing chemical fertilizers and to boost mineral absorption by the plant (Rathore et al., 2009; Singh et al., 2016). Seaweed extracts have been proven to have extremely low carbon footprints (Ghosh et al., 2015; Anand et al., 2018) and upon application to crops can lower global warming potential per unit of yield obtained (Sharma et al., 2017; Singh et al., 2018; Singh et al., 2023), thus serving the purpose of cleaner production.

In order to reduce the cost of corm production on a commercial scale and make corms available for area development, it is now clear that studying the behaviour of saffron corms for the daughter corm generation is of the utmost importance. Since there are few studies on the effect of seaweed on the saffron crop mainly focusing on stigma yield and quality (Behdani et al., 2020; Deh-Arbab et al., 2020), however, studies related to the effect of seaweed extracts in enhancing saffron corm production are lacking which is the most expensive input in the growth of saffron. Therefore, the objective of the present study was to examine the effect of the method of application of seaweed extract on saffron for optimum growth, corm production, biochemical and nutrient composition of saffron.

## 3 Materials and methods

### 3.1 Experimental site

**Figure 1:**
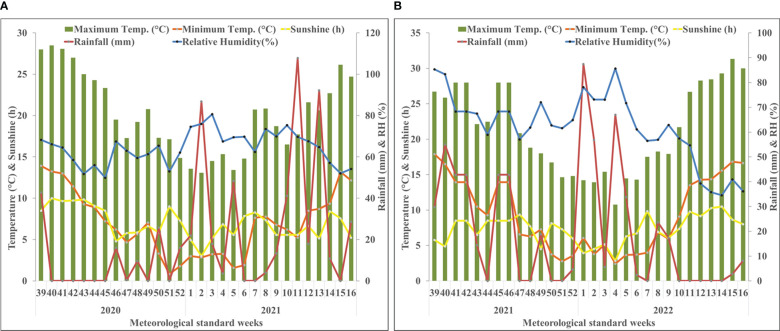
Weekly mean meteorological data during the period of the experiment at Palampur, Himachal Pradesh, India. **(A)** weather data of the first cropping year (2020–2021); **(B)** weather data of the second cropping year (2021–2022). The starting date of 39th meteorological standard week (MSW) and closing date of 16th MSW are 24th September and 22nd April, respectively. BSS, bright sunshine hours; RH, relative humidity.

The investigation was carried out at the experimental farm of CSIR – Institute of Himalayan Bioresource Technology (CSIR-IHBT) situated at an altitude of 1400 m amsl (above mean sea level) (32°6’29” N, 76°33’33” E) during the 2020-21 and 2021-22 cropping season. Weather data, *viz.*, temperature, humidity, and rainfall, were deduced from the local, regional weather station situated at Palampur, Himachal Pradesh during 2020-21 (**Figure 1A**) and 2021-22 (**Figure 1B**). The mean maximum temperature was 20.3°C ranging from 7.5°C to 29.5°C and 21.26°C ranging from 6°C to 32°C while the mean minimum temperature was 7.1°C ranging from -1°C to 18°C and 9.3°C ranging from 0°C to 20°C during 2020-21 and 2021-22, respectively. The mean relative humidity was 64.18% and 60.84% during 2020-21 and 2021-22, respectively. Rainfall of 623.8 and 450.4 mm was obtained overall throughout the crop growing seasons of 2020-21 and 2021-22, respectively. The soil in the test plot had a sandy clay loam texture, with an acidic reaction (pH 5.2) electrical conductivity of 0.14 m mhos/cm, and low organic carbon (0.45%). The available nitrogen was low (157.3 kg/ha), while phosphorus (19.1 kg/ha) and potassium (239.7 kg/ha) were medium in range.

### 3.2 Experimental details

A randomized block design (RBD) was used to study the method of application of seaweed extract in saffron. The seaweed extract used was AQU-ICSP (made by a physical mixture of 80% Kappaphycus aqueous extract + 20% Sargassum aqueous extract on a dry weight basis) procured from M/s Aquagri Processing Pvt Ltd., New Delhi, India. The composition of Kappaphycus and Sargassum aqueous extract was detailed in **Table 1**. The heavy metal contents of Cr, Pb, Co, As, Cd were well below the prescribed limits recommended for biostimulants under Fertilizer Control Order guidelines 2021 (Vaghela et al., 2023). Five treatments, *viz*., T1 (Control); T2 (Corm dipping in 5% seaweed extract solution); T3 [Foliar spray of 5% seaweed extract solution at 30, 45, 60, 75 days after sowing (DAS)]; T4 (Drenching with 5% seaweed extract solution in the soil after corm sowing at 30, 45, 60, 75 DAS) and T5 (corm dipping + foliar spray with 5% seaweed extract solution at 30, 45, 60, 75 DAS) with five replications were used to assess the effect of different treatments on the growth and corm production of saffron. A total of 25 beds, each measuring 1 m ×1 m (1 m^2^) were prepared. The corms weighing 8-10 g were planted at a depth of 10-12 cm spaced between 20 cm X 10 cm apart during the last week of September in both cropping years. Beds were raised to a height of 20 cm to drain extra water from the beds during rains since Palampur is a rain-fed area. To ward off the fungal infestation, the corms were treated with fungicide (carbendazim 50% WP) solution for half an hour and were dried in the shade. Hand weeding was used to control weeds at intervals of 15 days and as needed to maintain a weed-free crop throughout the growing season.

**Table 1:** Composition of Kappaphycus and Sargassum aqueous extracts.

Composition	Kappaphycus aqueous extract	Sargassum aqueous extract
Nitrogen (N)	0.33%	0.1%
Phosphorus (P)	0.08%	0.95%
Potassium (K)	30.24%	9.7%
Sulphur (S)	3.3%	3.2%
Calcium (Ca)	1.3%	1.1%
Magnesium (Mg)	1%	0.8%
Boron (B)	21.7 ppm	57 ppm
Iron (Fe)	202.5 ppm	83 ppm
Copper (Cu)	1 ppm	3.2 ppm
Zinc (Zn)	2 ppm	4.4 ppm
Gibberellic acid (GA_3_)	14 ppm	0 ppm
Indole acetic acid	1.1 ppm	0.4 ppm
Zeatin	0.6 ppm	4 ppm
Total polyphenols	1.2 g Gallic acid equivalent 100 g^-1^	1.0 g Gallic acid equivalent 100 g^-1^
Total flavonoid	69 mg Quercetin equivalent 100 g^-1^	65 mg Quercitine equivalent 100 g^-1^

(Source: Vaghela et al., 2023).

### 3.3 Data collection

Growth parameters, *viz.*, plant height, number of leaves, and leaf lengths were recorded from 5 plants/plot at different growth stages (40 and 90 DAS) of saffron. The data on dry matter partitioning (mg/plant), i.e. dry weight of stem, leaf, root, corm, and total dry weight, were recorded at 40 and 90 DAS. For corm production data, the number of daughter corms was calculated at 45 and 90 DAS, along with the total number and weight of corms/m^2^ at harvest. Corm number was further categorized based on different grades like corm weight below 5 g, 5.1-8.0 g, 8.1-12.0 g, and more than 12 g.

### 3.4 Soil analysis

Before the first crop season, soil samples from the experimental plot were collected, dried at room temperature, and then passed through a 30 mesh panel. P_2_O_5_ and K_2_O availability were calculated using Mehlich No. 3’s technique (Mehlich, 1984). For the analysis of the available nitrogen and organic carbon, the macro Kjeldahl method and the Walkley and Black methods, respectively, were applied (Black, 1965). The soil’s pH and texture were assessed using a pH meter and the hydrometer method (Black, 1965).

### 3.5 Total chlorophyll, carotenoid content, and photosynthetic rate

For the analysis of chlorophyll and carotenoids, one-gram leaf fresh samples were collected at 90 DAS in 2021–2022 and homogenised with acetone (80%) before being centrifuged at 5000 rpm for 5 minutes. The absorbance (OD) of the supernatant was then measured at 663, 645, and 470 nm using a spectrophotometer (model T 90 + UV/vis, PG Instrument Ltd.). According to Arnon (1949), the following formulas were used to calculate the content of chlorophyll a, chlorophyll b, and carotenoids (mg/g):

Chlorophyll a=12.7(OD_663_)−2.69(OD_645_)

Chlorophyll b=22.9(OD_645_)−4.68(OD_663_)


$$\text{Carotenoids}=(1000\;\text{O}{\text{D}}_{470}-3.27\text{Chl}\;\text{a}-104\;\text{Chl}\;\text{b})/229$$

Photosynthetic measurement was also taken at 90 DAS during 2021-22, with the help of LI-6400 photosynthesis system (Li-COR, Lincoln, USA) details of which are mentioned in Kumar et al. (2020b).

### 3.6 Nutrient analysis of daughter corms

Corms from each experimental unit were gathered at the end of the second year in order to estimate various nutrients. Corm samples were grinded and sieved using a 0.7 mm sieve plate. The materials were digested with concentrated H_2_SO_4_ and concentrated HNO_3_ and perchloric acid for N and P, K analysis, respectively. Kel Plus nitrogen analyser, spectrophotometer (model T 90 + UV/vis, PG Instrument Ltd., UK), a flame photometer (model BWB XP, BWB Technologies UK Ltd., UK), and an atomic absorption spectrophotometer were used for the estimation of nutrients as per the procedure given by Prasad et al. (2006).

### 3.7 Statistical analysis

One-way ANOVA was used in a randomised block design (RBD) to analyse data obtained on several saffron production and growth characteristics. The values of the least significant difference (LSD) at *P* = 0.05 were multiplied by the standard errors of the means (SEM) to get the treatment variance. To assess the principal component analysis (PCA) and correlation, PAST3 (Paleontological Statistics Software Package for Education and Data Analysis version 3) software was employed (Hammer et al., 2001; Walia et al., 2021).

## 4 Results

### 4.1 Growth parameters

**Figure 2:**
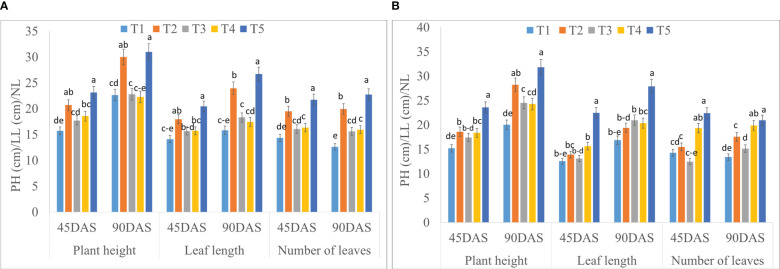
Effect of different methods of seaweed extract application on growth parameters, *viz.*, plant height (PH), leaf length (LL) and number of leaves (NL) of saffron at different growth intervals 45DAS and 90DAS during 2020-21 **(A)** and 2021-22 **(B)**. Results are represented as the means of five replications (n=5) ± SE, bars with different letters are significantly different at *P* = 0.05. DAS: Days after sowing. T1: Control; T2: Corm dipping in 5% seaweed extract; T3: (Foliar spray of 5% seaweed extract at 30, 45, 60, 75 DAS); (Drenching with 5% seaweed extract in the soil after corm sowing at 30, 45, 60, 75 DAS); T5 (corm dipping + foliar spray with 5% seaweed extract at 30, 45, 60, 75 DAS).

The growth parameters of saffron were significantly influenced by the application of seaweed extract (**Figure 2**; **Supplementary Table S1**). Statistically higher plant height at 45 DAS (23.15 and 23.55 cm) and 90 DAS (30.99 and 31.82 cm) were recorded in treatment T5 (Corm dipping + foliar spray @ 5% seaweed extract) during 2020-21 and 2021-22, respectively, compared to control. Treatment T5 remained in line with T2 at 45 and 90 DAS during both years, except at 45 DAS during 2021-22. T5 recorded 47.35 and 55.13% higher plant height at 45 DAS while 36.94 and 58.94% higher at 90 DAS during 2020-21 and 2021-22, respectively when compared to control. Leaf length was also found to be significantly higher in T5 at 45 and 90 DAS as compared to control during both years; however, it remained at par with T2 at 45 DAS during 2020-21. A significantly higher number of leaves were found in T5 at both the dates (45 and 90 DAS) of 2020-21 and 2021-22, while this treatment remained in line with T4 at 45 and 90 DAS during 2021-22. The number of leaves in T5 treatment was 51.63 and 56.97% higher at 45 DAS while 80.39 and 56.19% higher at 90 DAS during 2020-21 and 2021-22, respectively when compared to control.

### 4.2 Dry matter partitioning

**Figure 3:**
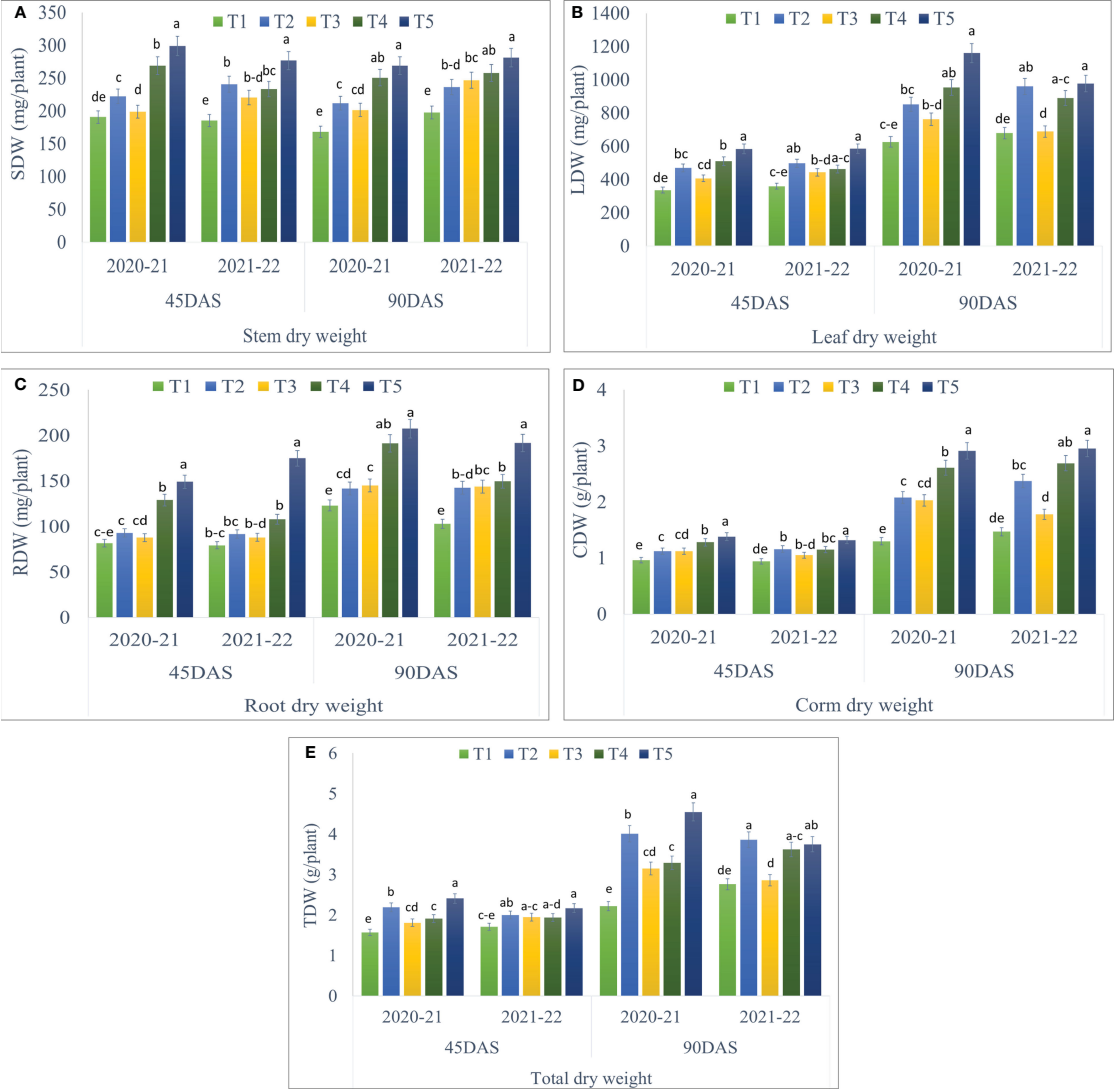
Effect of different methods of seaweed extract application on dry weight of different plant parts, *viz.*, **(A)** stem dry weight (SDW), **(B)** leaf dry weight (LDW), **(C)** root dry weight (RDW), **(D)** corm dry weight (CDW) and **(E)** total dry weight (TDW) of saffron at different growth intervals 45DAS and 90DAS during 2020-21 and 2021-22. Results are represented as the means of five replications (n=5) ± SE, bars with different letters are significantly different at *P* = 0.05. DAS: Days after sowing. T1: Control; T2: Corm dipping in 5% seaweed extract; T3: (Foliar spray of 5% seaweed extract at 30, 45, 60, 75 DAS); (Drenching with 5% seaweed extract in the soil after corm sowing at 30, 45, 60, 75 DAS); T5 (corm dipping + foliar spray with 5% seaweed extract at 30, 45, 60, 75 DAS).

Seaweed extract application showed significant differences in all the parameters of dry matter partitioning during both the years of study and the data has been given in **Figure 3**. Treatment T5 (Corm dipping + foliar spray @ 5% seaweed extract) recorded significantly higher stem dry weight at 45 (298.91 and 276.70 mg/plant) and 90 DAS (268.79 and 280.93 mg/plant) during 2020-21 and 2021-22, respectively as compared to control. At 90 DAS, treatment T5 remained statistically at par with T4 during both years (**Figure 3A**; **Supplementary Tables S2**). The dry weight of leaves per plant was also recorded statistically higher in T5 as compared to the control but was in line with T4 at 45 and 90 DAS during both years, except at 45 DAS during 2020-21. T5 recorded 73.74 and 62.93% higher leaf dry weight at 45 DAS, while 85.26 and 43.94% higher at 90 DAS during 2020-21 and 2021-22, respectively, when compared to control (**Figure 3B**; **Supplementary Tables S2**). Significantly higher root dry weight per plant was recorded in T5 at 45 and 90 DAS during both the year than in control, however, it remained in line with T4 at 90 DAS during 2020-21 (**Figure 3C**; **Supplementary Table S2**). Corm dry weight per plant was also recorded significantly higher in T5 with 43.05 and 40.19% higher corm dry weight at 45 DAS, while 123.43 and 100.14% higher at 90 DAS during 2020-21 and 2021-22, respectively, when compared to control (**Figure 3D**; **Supplementary Table S3**). Treatment T5 remained statistically at par with T4 at 90 DAS during 2021-22. Statistically higher total dry weight per plant was recorded in T5 as compared to control during both years. T5 remained in line with T2, T3, and T4 at 45 DAS, while T2 and T4 at 90 DAS during 2021-22 (**Figure 3E**; **Supplementary Table S3**).

### 4.3 Corm production

**Figure 4:**
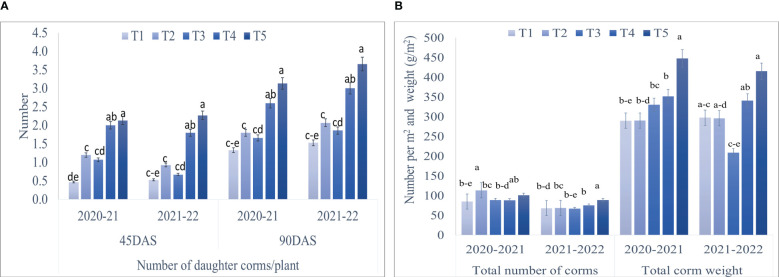
Effect of different methods of seaweed extract application on corm production, *viz.*, **(A)** number of daughter corms/plant, **(B)** total number of corms and total corm weight (per m^2^) of saffron during 2020-21 and 2021-22. Results are represented as the means of five replications (n=5) ± SE, bars with different letters are significantly different at *P* = 0.05. DAS: Days after sowing. T1: Control; T2: Corm dipping in 5% seaweed extract; T3: (Foliar spray of 5% seaweed extract at 30, 45, 60, 75 DAS); (Drenching with 5% seaweed extract in the soil after corm sowing at 30, 45, 60, 75 DAS); T5 (corm dipping + foliar spray with 5% seaweed extract at 30, 45, 60, 75 DAS).

**Figure 5:**
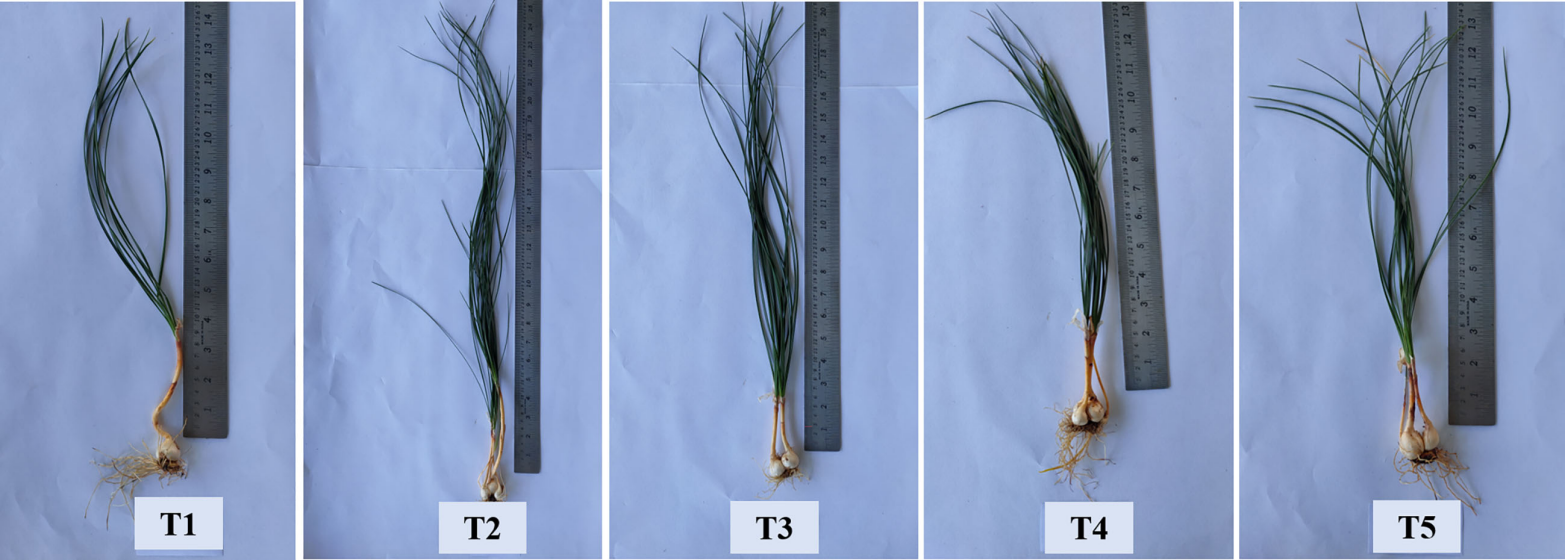
Treatment-wise representative plants of saffron showing effect of different methods of seaweed extract application on corm production at 90 DAS. T1: Control; T2: Corm dipping in 5% seaweed extract; T3: (Foliar spray of 5% seaweed extract at 30, 45, 60, 75 DAS); (Drenching with 5% seaweed extract in the soil after corm sowing at 30, 45, 60, 75 DAS); T5 (corm dipping + foliar spray with 5% seaweed extract at 30, 45, 60, 75 DAS).

Maximum corm production parameters, *viz.*, the number of daughter corms per plant, the total number of corms/m^2^, and total corm weight (g/m^2^) showed significant results during both the years (**Figures 4**, **5**). A significantly higher number of daughter corms per plant were observed in T5 (Corm dipping + foliar spray @ 5% seaweed extract) compared to control at 45 and 90 DAS during both years. Treatment T5 remained statistically at par with T4 (Drenching @ 5% seaweed extract after corm sowing) during both years except at 90 DAS during 2020-21. At 90 DAS, treatment T5 recorded 135.33 and 139.21% higher number of daughter corms as compared to control during 2020-21 and 2021-22, respectively (**Figure 4A**; **Supplementary Table S4**). During 2020-21, the total number of corms/m^2^ was recorded significantly higher in T2 (Corm dipping @ 5% seaweed extract) compared to control, followed by T5. However, during 2021-22, treatment T5 (Corm dipping + foliar spray @ 5% seaweed extract) recorded a significantly higher total number of corms/m^2^, closely followed by T4 when compared with the control. Treatment T2 and T5 recorded 33.64 and 30.20% higher total number of corms/m^2^ as compared to control during 2020-21 and 2021-22, respectively. Total corm weight (g/m^2^) was recorded significantly higher in T5 during both years, with 54.43 and 39.55% respectively, when compared with control (**Figure 4B**; **Supplementary Table S4**).

A significant increase in the number of daughter corms/m^2^ was observed at harvest for corms categorized according to their weight (**Table 2**). Daughter corms weighing more than 12 g were found significantly higher in number in T5 compared to control during both years. Daughter corms of 12 g weight were 190.90 and 244.44% higher in T5 treatment during 2020-21 and 2021-22, respectively than in control. Treatment T5 also recorded a significantly higher number of daughter corms of 8-12 and 5-8 g categories during 2020-21 and 2021-22 when compared with other seaweed extract treatments. However, corms weighing less than 5 g were found statistically higher in T2 during 2020-21. During 2021-22, no significant effect was observed for the number of daughter corms weighing less than 5 g.

**Table 2:** Effect of different methods of seaweed extract application on number of daughter corm of saffron as per different categories at harvest.

Treatment	Weight wise categories of daughter corms at harvest (number/m^2^)									
		>12 g		8-12 g		5-8 g		<5 g		
	2020-21	2021-22	2020-21	2021-22	2020-21	2021-22	2020-21	2021-22		
T1	2.2 ± 0.97^bc^	1.8 ± 0.37^cd^	4.6 ± 1.29^b-d^	5.6 ± 0.51^c-e^	8.6 ± 2.02^b-e^	12.2 ± 0.66^bc^	69.6 ± 5.64^b^	48.6 ± 3.78		
T2	2.6 ± 0.51^b^	2.8 ± 0.37^c^	4.4 ± 1.29^b-e^	6.0 ± 0.55^bc^	12.0 ± 1.38^b^	10.2 ± 0.49^b-d^	94.6 ± 2.04^a^	49.8 ± 2.99		
T3	2.2 ± 0.74^bc^	1.6 ± 0.51^c-e^	6.0 ± 1.05^b^	7.4 ± 0.51^b^	11.6 ± 1.21^bc^	9.0 ± 1.27^b-e^	69.0 ± 5.73^b-d^	49.0 ± 2.26		
T4	2.2 ± 0.37^bc^	4.6 ± 0.51^b^	5.4 ± 1.03^bc^	5.8 ± 0.37^b-d^	11.0 ± 1.10^b-d^	12.8 ± 0.97^b^	69.4 ± 5.58^bc^	51.6 ± 1.99		
T5	6.4 ± 0.51^a^	6.2 ± 0.58^a^	9.2 ± 1.02^a^	9.6 ± 0.51^a^	17.0 ± 1.30^a^	18.4 ± 1.44^a^	68.4 ± 9.60^b-e^	54.6 ± 1.86		
SEm(±)	0.55	0.41	0.83	0.53	1.41	1.12	6.13	2.78		
LSD (*P*=0.05)	1.68	1.24	2.53	1.62	4.26	3.41	18.56	NS		

SEm(±), Standard Error of Mean; LSD, Least Significant Difference. T1: Control; T2: Corm dipping in 5% seaweed extract; T3: (Foliar spray of 5% seaweed extract at 30, 45, 60, 75 DAS); (Drenching with 5% seaweed extract in the soil after corm sowing at 30, 45, 60, 75 DAS); T5 (corm dipping + foliar spray with 5% seaweed extract at 30, 45, 60, 75 DAS). Superscript letters represent : Means within each column with similar letter are not significantly different at the 5% probability level. NS, Non significant.

### 4.4 Principal component analysis

**Figure 6:**
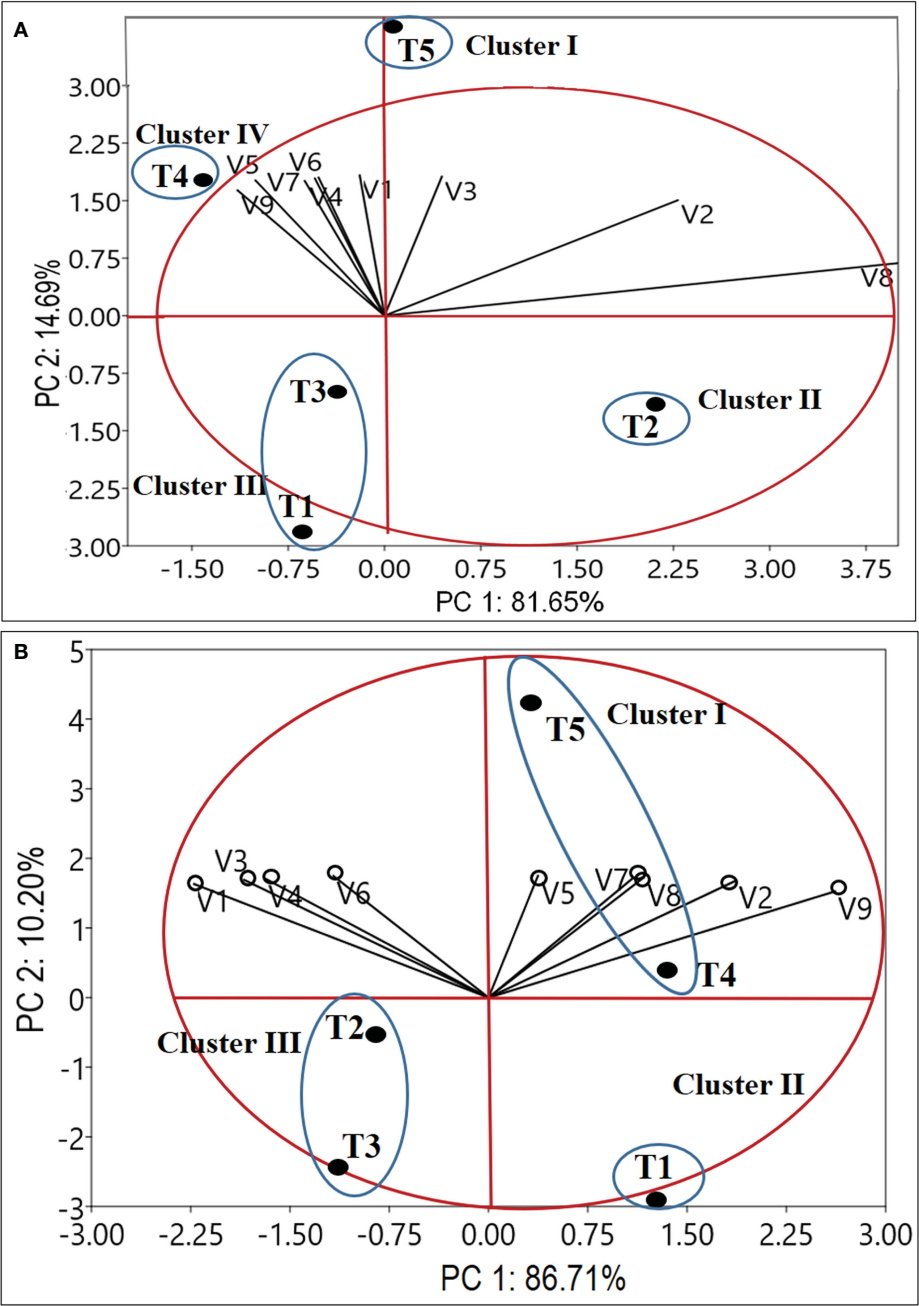
Bi-plot of principal components based on mean value of growth and yield attributes. Factor1 and Factor 2 explain 96.34 and 96.91% of the data variation for 2020-21 **(A)** and 2021-22 **(B)**, respectively. Figure a, and b represents variable vector distributions and the treatment distributions. V1 -Plant height (cm); V2-Number of leaves/plant; V3-Leaf dry weight (mg/plant); V4-Stem dry weight (mg/plant); V5-Root dry weight (mg/plant); V6-Corm dry weight (mg/plant); V7-Number of daughter corms/plant; V8-Total number of corms/m^2^; V9-Total corm weight (g/m^2^); T1 – Control; T2 - Corm dipping in sea weed solution (5%); T3 - Foliar spray @ 5%; T4 – Drenching @ 5% after corm sowing; T5 – Corm dipping + foliar spray @5% sea weed).

The set of nine variables for the year 2020-2021 and 2021-2022 were used in the principal component analysis (PCA). According to the information shown in **Figure 6**, PC1 and PC2 account for 96.34% and 96.71% variations for the years 2020–21 and 2021–22, respectively. The links between the variables in the first two components (PC1 and PC2) space are shown in **Figure 6**, which also shows how much each variable contributed to the main components in each year. During 2020-21, except for the number of leaves/plant (V2), leaf dry weight (V3), and the total number of corms/m^2^ (V8), all variables [Plant height (V1cm); stem dry weight (V4); root dry weight (V5); corm dry weight (V6); the number of daughter corms/plant (V7) and total corm g/m^2^ (V9)] are situated in PC1’s negative coordinate. The PCA separated treatments into four distinct clusters. Cluster I, exhibited a higher value of V1 (23.15 cm), V2 (21.73), V3 (583.41 mg), V4 (298.91 mg), V5 (149.00 mg), V6 (1385.14 mg), V7 (2.13) andV9 (447.62 g/m^2^). Cluster II exhibited the highest value of V8 (101.00), while cluster III represents the lower range of V1 (15.71-17.71cm), V2 (14.33-16.06), V3 (335.79-405.92 mg), V4 (190.54-198.87 mg), V5 (81.68-87.71 mg), V6 (968.25-1125.57mg), V7 (0.47-1.07) and V9 (289.84-330.20g/m^2^). Cluster IV represents an intermediate range of most of the variables and the lowest range of V8 (88.00). The treatment T5 (Corm dipping + foliar spray @ 5% seaweed extract) was separated by PC1 and PC2 in the PCA bi-plot (**Figure 6A**), and it was positioned in the positive coordinate of both PCs. In contrast, the treatments T1 and T3 are grouped together. The data shown in **Figures 2**–**5** were supported by the PCA bi-plots (**Figure 6**), which explained the strong connections between the key variables for T5.

During 2021-22, the PCA bi-plot (**Figure 6B**) shows variables V2, V5, V7, V8, and V9 in positive coordinates and V1, V3, V4, and V6 in negative coordinates. The PCA separated treatments into three distinct clusters. Cluster I, exhibited the highest range of all the nine variables. Cluster II exhibited the lowest range of all the variables, while cluster III exhibited an intermediate range of all the nine variables. The treatment T5 (Corm dipping + foliar spray @ 5% seaweed extract) and T4 were divided along with PC1 and PC2, in the case-distribution-plot (**Figure 6B**). Thus, the overall PCA output indicates that T5 (Corm dipping + foliar spray @ 5% seaweed extract) represents the best treatment distinctly differentiated from the rest of the treatments during both 2020-21 and 2021-22. The first two PCs were most informative, with Eigen values 7.34 and 1.32 for 2020-21 and 8.10 and 1.02 for 2021-22.

### 4.5 Correlation analysis

**Figure 7:**
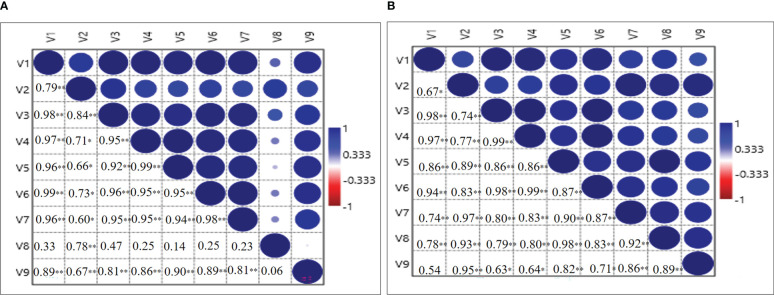
Correlation analysis between growth and corm parameters for the year 2020-21 **(A)** and 2021-22 **(B)**, respectively. V1-Plant height (cm); V2-Number of leaves/plant; V3-Leaf dry weight (mg/plant); V4-Stem dry weight (mg/plant); V5-Root dry weight (mg/plant); V6-Corm dry weight (mg/plant); V7-Number of daughter corms/plant; V8-Total number of corms/m^2^ and V9-Total Corm g/m^2^. The mean values of five biological replicates of the corresponding treatments (5) were used, ∗ and ∗∗ indicate that the corresponding values are significant at *P* ≤ 0.05 and *P* ≤ 0.01, respectively.

The correlation matrix among the variables plant height (V1), number of leaves/plant (V2), leaf dry weight (V3), stem dry weight (V4), root dry weight (V5), corm dry weight (V6), number of daughter corms/plant (V7), the total number of corms/m2 (V8) and total corm weight g/m^2^ (V9) was also established during 2020-21 and 2021-22 (**Figure 7**). During 2020-21, a significant positive (*P* = 0.01) correlation was found between V1 and all the other variables except V8, however, during 2021-22 significantly positive (*P* = 0.01) correlation was recorded with V3, V4, V5, V6, V7 and V8. V2 also showed a positive correlation with V1 but at *P* = 0.05 significance level. A significant (*P* = 0.01) positive correlation was found between V3 and variables V1, V2, V8, V9; however positive correlation at P = 0.05 significance level was observed for V4, V5, V6 and V7 during 2020-21 (**Figure 7A**). During 2021-22, a significantly positive (*P* = 0.01) correlation was found with all the variables. Significant and positive correlations of V4, V5, V6, and V7 were observed with all the other variables during both years except V8 during 2020-21. During 2020-21, V8 only showed a significant and positive correlation with V2 and V9 at *P* = 0.01 significance level, while during 2021-22, it was seen with all the variables. V9 showed a positive correlation at *P* = 0.01 significance level with V1, V2, V3, V4, V5, V6, V7 during 2020-21 and with V2, V5, V7, V8 during 2021-22 (**Figure 7B**).

### 4.6 Biochemical and nutrient composition of saffron

**Figure 8:**
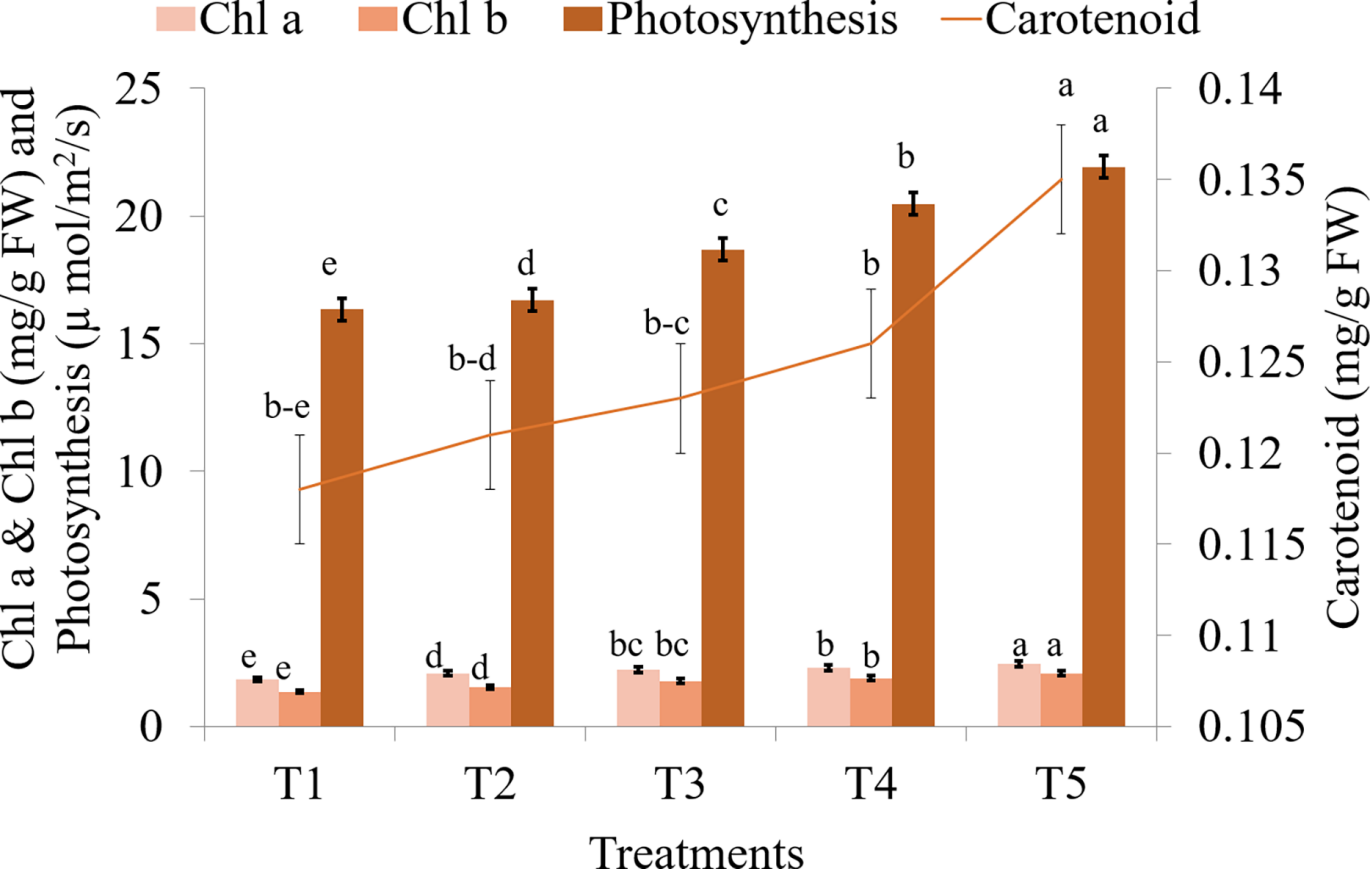
Effect of different methods of seaweed extract application on chlorophyll and photosynthesis of saffron during 2021-22. Results are represented as the means of five replications (n=5) ± SE, bars with different letters are significantly different at *P* = 0.05. DAS: Days after sowing. T1: Control; T2: Corm dipping in 5% seaweed extract; T3: (Foliar spray of 5% seaweed extract at 30, 45, 60, 75 DAS); (Drenching with 5% seaweed extract in the soil after corm sowing at 30, 45, 60, 75 DAS); T5 (corm dipping + foliar spray with 5% seaweed extract at 30, 45, 60, 75 DAS).

**Figure 9:**
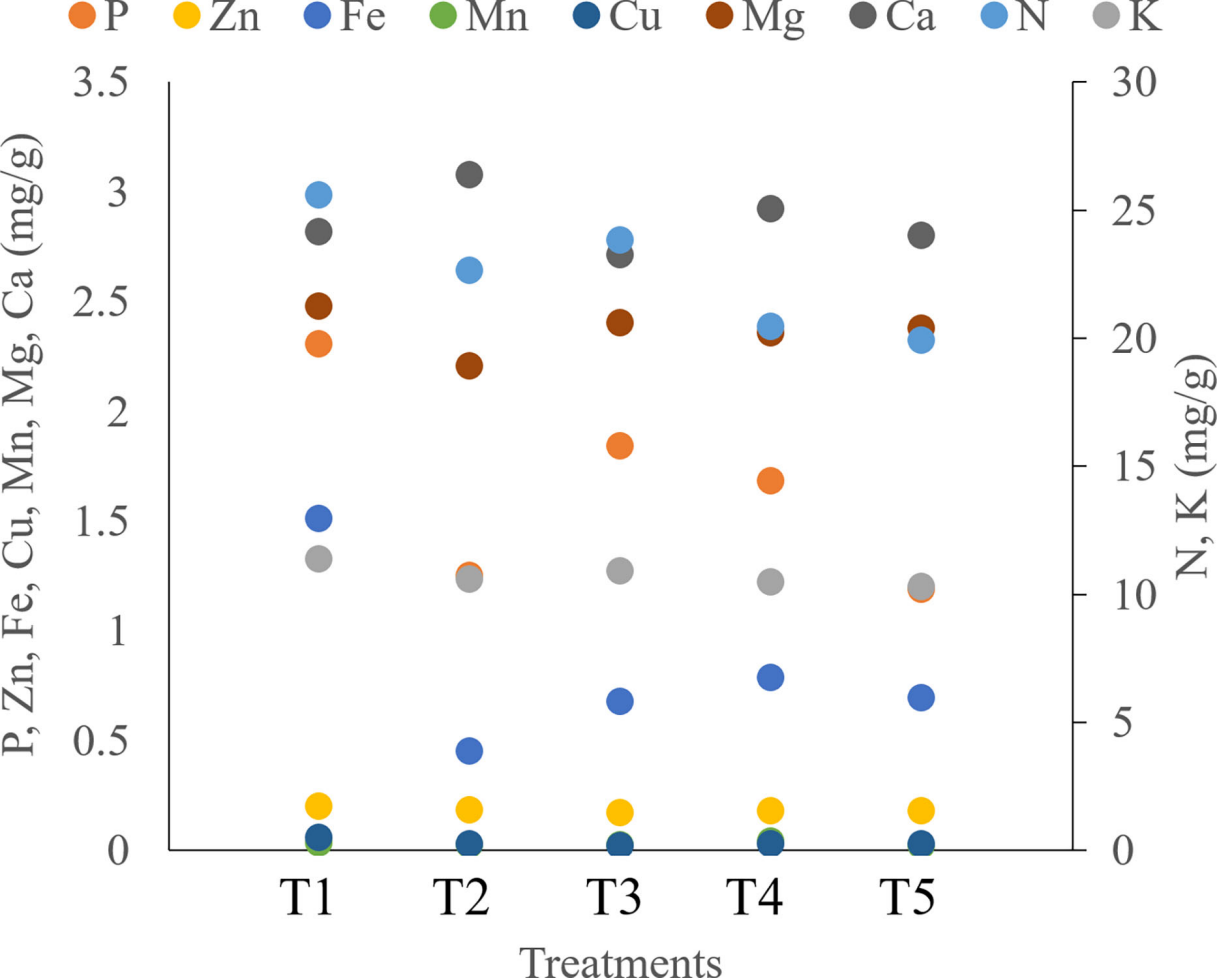
Effect of different methods of seaweed extract application on nutrient composition of saffron corms obtained during harvest.

Seaweed extract treatment significantly enhanced the levels of chl a, chl b, and carotenoids in saffron leaves when compared to the control (**Figure 8**; **Supplementary Table S5**). Among seaweed extract treatments, T5 recorded the maximum value of both chlorophyll and carotenoids. Additionally, it was found that plants treated with seaweed extract have much increased photosynthetic rates, with T5 treatment having the highest rates. In the saffron corms, control recorded significantly higher levels of N, P, K, Zn, Fe, Cu, and Mg when compared to seaweed extract treatments (**Figure 9**; **Supplementary Table S6**). Macronutrients N, P, and K were found to be significantly lower in treatment T5, which was 22.23, 48.69, and 9.51% lower, respectively, than in the control. Zn and Cu were found lowest in T3 while Fe and Mg were in T2. Mn and Ca were found significantly higher in treatment T4 and T2, respectively, when compared with the control. Mn was found lowest in T5, T3, and T2, while Ca was in T3.

## 5 Discussion

The potential use of seaweed extracts in crop cultivation to increase biomass yield and produce quality has received extensive research. Seaweed extract, when applied to saffron plants (Corm dipping + foliar spray @ 5% seaweed extract) results in significantly higher growth parameters (**Figure 2**). Improved growth parameters by the usage of seaweed extract may be caused by the content of various phytohormones (Colapietra and Alexander, 2006). Auxins, cytokinin, gibberellins, abscisic acid, ethylene, and auxins are the main phytohormones found in seaweed extracts and are responsible for cell division, elongation of plant tissue growth, and apical dominance (Nirmani Kularathne et al., 2021). Al-Saad and Al-zubaidi (2021) recorded higher plants with a maximum number of leaves when sprayed with Acadian seaweed extract. Higher leaf length of saffron was also reported by Behdani et al. (2020) when the highest concentration of Acadian seaweed extract was applied to saffron plants through a foliar spray. The current findings were consistent with the findings made by Sridhar and Rengasamy (2010) and Ibrihem (2015) who used seaweed extract to treat *Tagetes erecta* L. and *Calendula officinalis* L., respectively, with effective results in terms of plant height and leaf number. Corm dipping along with foliar spray treatment operates better in saffron plants with immediate mobility of phytohormones and nutrients because of their direct engagement with plant tissues and fast absorption rate through the foliar application, along with the adsorption by soil particles through corm dipping (Ali et al., 2016; Ali et al., 2021a). A significantly higher dry weight of leaf, stem, root, and corms of saffron was also observed in our study with the application of the seaweed extract (**Figure 3**). These results were in line with the correlation analysis performed where significant and positive correlation values were observed between growth parameters and dry weight parameters of saffron. Behdani et al. (2020) and Deh-Arbab et al. (2020) recorded similar findings with higher dry leaf and petal weight of saffron when treated with seaweed extract. Sunflower plant species treated with a 5% dilution concentration of marine algae also provided the maximum fresh and dry mass of the whole plant (Nirmani Kularathne et al., 2021). Some researchers have highlighted the significance of seaweed extract in enhancing the plant biomass of various crops by increasing the soil ability to absorb nutrients (Turan and Kose, 2004; Al-Hamzawi, 2019).

Seaweed extract application also enhanced the corm production when both corm dipping and foliar spray of 5% concentration were applied to the saffron plants (**Figure 4** and **Table 2**). Enhancement in the growth parameters might have led to higher corm production which is also supported by a significant positive correlation observed in our findings between these growth and corm production parameters. Small amounts of phytohormones like auxins that are present in the seaweed extracts and numerous stimulatory mechanisms that are activated in the plant system as a result of treatment with these extracts may explain the better rooting architecture (Ali et al., 2021b). Besides the presence of hormones, the seaweed extracts in the present study also contained polyphenols and flavonoids which also has known positive effect on plants (Vaghela et al., 2022). One of the constituents of the present formulation used is an extract from the seaweed *Kappaphycus alvarezii*, which has been found to modulate a number of genes and transcription factors towards favourably increasing biomass and yield in maize upon foliar application or drenching (Kumar et al., 2020a; Trivedi et al., 2021b). Our findings were supported by the studies of Al-Saad and Al-zubaidi (2021), reporting a higher number and weight of corms from the plants treated with Acadian seaweed extract. Behdani et al. (2020) also observed that the application of seaweed extract at higher concentrations resulted in the maximum weight of daughter corms. The application of seaweed extract considerably increased the amount of chl a, chl b, carotenoid content, and photosynthetic rate in the current study (**Figure 8**). Similarly, seaweed extract application on onion plants enhanced the chlorophyll and carotenoid content in onion bulbs (Szczepanek et al., 2017; Gupta et al., 2021). The increase in photosynthetic pigments suggests that these plants are more physiologically active than control plants and can create more photosynthetic assimilates, resulting in higher photosynthetic rates. The production of more leaves is important for greater corm or bulb growth (Abdissa et al., 2011). The considerable increase in corm size and quantity in the current study may be attributable to the seaweed-treated plants having much higher chlorophyll content and photosynthetic rate. Plants with seaweed extract application had a lower amount of N, P, K, Zn, Fe, Cu, and Mg in the saffron corms as compared to the control (**Figure 9**). It might be attributed to the efficient uptake and use of these nutrients, which led to a notable rise in the content of pigments, the number of leaves, the number of daughter corms, and the corm weight. Onion leaves and bulbs recorded the lowest levels of N, K, and Mg, according to Gupta et al. (2021), when plants were treated with seaweed extract, while the highest nutrient uptake was observed in control. By promoting root growth, seaweed extracts increase nutrient absorption (Mancuso et al., 2006). Ertani et al. (2018) reported that maize leaves were able to absorb considerably more nutrients than the controls when *Ascophyllum nodosum* and *Laminaria* spp. extracts were applied.

## 6 Conclusion

Generally, the benefits of seaweed extract-based biostimulants to the environment and crop production support their prescription for use in various cropping systems. Seaweed extract of Kappaphycus and Sargassum seaweed combination, when applied to the saffron crop, showed better results in all aspects of growth, dry weight, nutrient uptake, and saffron corm production. Simultaneous application of seaweed extract before and after sowing (T5: Corm dipping + foliar spray @ 5% seaweed extract) recorded a higher number of daughter corms and a higher weight of corms per m^2^. Nutrients in corms were found highest in control and lowest in T5 because of efficient uptake and use of these nutrients in increasing corm production, making it a feasible alternative to limiting the application of conventional fertilizers and attenuating the effects on the environment, leading to a cleaner environment. The studies indicate a need for additional investigation on the application of different seaweed extracts with different concentrations along with PGPR to see the synergistic effect on yield and quality of saffron production.

## Data availability statement

The original contributions presented in the study are included in the article/**Supplementary Material**. Further inquiries can be directed to the corresponding author.

## Author contributions

NC: Formal analysis, data observation, literature search, writing; DK: Formal analysis, data observation; SW: Data compilation, statistical analysis, data curation, data presentation, literature search, and writing - original draft; AG: Data curation, Validation, Writing - review & editing; PV: Data curation, Validation, Writing - review & editing and RK: Conceptualization, Methodology Project administration, Supervision, Data curation, Validation. Writing - review & editing. All authors contributed to the article and approved the submitted version
