# Supplementary material for: Biostimulant enhances growth and corm production of saffron (Crocus sativus L.) in non-traditional areas of North western Himalayas
Source: Front Plant Sci. 2023 Feb 15;14:1097682. doi: 10.3389/fpls.2023.1097682 (PMC9975171; doi:10.3389/fpls.2023.1097682)
Supplement: Supplementary file 1 [file DataSheet_1.docx]

Table S1. Effect of different methods of seaweed extract application on growth parameters of saffron at different growth intervals 45DAS and 90DAS.

| Treatment | Plant height (cm) | | | | Leaf length (cm) | | | | Number of leaves/plant | | | |
| --- | --- | --- | --- | --- | --- | --- | --- | --- | --- | --- | --- | --- |
|  | 45DAS | | 90DAS | | 45DAS | | 90DAS | | 45DAS | | 90DAS | |
|  | 2020-21 | 2021-22 | 2020-21 | 2021-22 | 2020-21 | 2021-22 | 2020-21 | 2021-22 | 2020-21 | 2021-22 | 2020-21 | 2021-22 |
| T1 | 15.7±0.64^de^ | 15.1±0.56^de^ | 22.6±2.76^cd^ | 20.0±1.33^de^ | 14.1±0.88^c-e^ | 12.5±0.38^b-e^ | 15.8±0.80^c-e^ | 16.8±1.42^b-e^ | 14.3±0.80^de^ | 14.2±0.91^cd^ | 12.6±0.46^c^ | 13.4±0.51^de^ |
| T2 | 20.6±1.99^ab^ | 18.6±1.71^b^ | 29.9±2.44^ab^ | 28.2±1.15^ab^ | 17.9±0.56^ab^ | 13.9±0.78^bc^ | 23.9±1.67^b^ | 19.3±0.75^b-d^ | 19.4±0.91^b^ | 15.4±0.40^c^ | 19.9±0.79^b^ | 17.5±1.04^c^ |
| T3 | 17.7±0.95^cd^ | 17.4±0.65^b-d^ | 22.8±1.35^c^ | 24.5±0.15^c^ | 15.6±1.34^b-d^ | 13.0±0.59^b-d^ | 18.2±0.79^c^ | 20.9±1.36^b^ | 16.0±0.31^cd^ | 12.4±0.89^c-e^ | 15.6±0.49^cd^ | 15.1±0.39^d^ |
| T4 | 18.5±0.86^bc^ | 18.3±0.95^bc^ | 22.2±0.98^c-e^ | 24.2±1.24^cd^ | 15.7±1.19^bc^ | 15.6±1.83^b^ | 17.4±0.88^cd^ | 20.3±1.68^bc^ | 16.3±0.61^c^ | 19.3±1.69^ab^ | 15.9±0.94^c^ | 19.8±0.92^ab^ |
| T5 | 23.1±1.93^a^ | 23.5±0.44^a^ | 30.9±1.64^a^ | 31.8±1.16^a^ | 20.4±0.86^a^ | 22.4±0.70^a^ | 26.7±1.58^a^ | 27.8±1.28^a^ | 21.7±0.09^a^ | 22.4±1.86^a^ | 22.7±0.99^a^ | 20.9±0.75^a^ |
| SEm(±) | 0.87 | 0.87 | 1.28 | 1.20 | 0.92 | 1.08 | 0.89 | 1.32 | 0.61 | 1.22 | 0.68 | 0.76 |
| LSD (*P*=0.05) | 2.63 | 2.64 | 3.88 | 3.63 | 2.78 | 3.28 | 2.68 | 4.00 | 1.83 | 3.69 | 2.06 | 2.29 |

Note: SEm(±): Standard Error of Mean; LSD: Least Significant Difference; DAS: Days after sowing. T1: Control; T2: Corm dipping in 5% seaweed extract; T3: (Foliar spray of 5% seaweed extract at 30, 45, 60, 75 DAS); (Drenching with 5% seaweed extract in the soil after corm sowing at 30, 45, 60, 75 DAS); T5 (corm dipping + foliar spray with 5% seaweed extract at 30, 45, 60, 75 DAS).

Table S2. Effect of different methods of seaweed extract application on dry weight of different plant parts of saffron at different growth intervals 45DAS and 90DAS.

| Treatment | Stem dry weight (mg/plant) | | | | Leaf dry weight (mg/plant) | | | | Root dry weight (mg/plant) | | | |
| --- | --- | --- | --- | --- | --- | --- | --- | --- | --- | --- | --- | --- |
|  | 45DAS | | 90DAS | | 45DAS | | 90DAS | | 45DAS | | 90DAS | |
|  | 2020-21 | 2021-22 | 2020-21 | 2021-22 | 2020-21 | 2021-22 | 2020-21 | 2021-22 | 2020-21 | 2021-22 | 2020-21 | 2021-22 |
| T1 | 190.5±6.55^de^ | 185.4±3.26^e^ | 168.1±2.84^e^ | 197.6±4.96^e^ | 335.7±18.35^de^ | 358.8±21.80^c-e^ | 626.1±57.63^c-e^ | 678.4±30.78^de^ | 81.6±6.09^c-e^ | 79.2±6.32^b-e^ | 123.1±1.88^e^ | 102.9±6.27^e^ |
| T2 | 221.8±6.58^c^ | 240.5±13.99^b^ | 211.7±6.93^c^ | 236.1±13.31^b-d^ | 468.5±52.41^bc^ | 497.1±69.04^ab^ | 850.8±69.16^bc^ | 959.4±66.91^ab^ | 92.8±3.06^c^ | 91.5±10.27^bc^ | 141.5±11.34^cd^ | 142.5±9.97^b-d^ |
| T3 | 198.8±9.88^d^ | 220.3±14.55^b-d^ | 201.4±18.23^cd^ | 246.4±3.98^bc^ | 405.9±10.52^cd^ | 442.8±56.65^b-d^ | 762.4±35.18^b-d^ | 687.7±67.66^d^ | 87.7±4.83^cd^ | 87.9±4.65^b-d^ | 145.0±9.05^c^ | 143.6±15.87^bc^ |
| T4 | 268.7±14.74^b^ | 233.3±9.91^bc^ | 250.5±11.78^ab^ | 257.5±5.54^ab^ | 509.0±43.98^b^ | 461.9±22.88^a-c^ | 952.8±39.96^ab^ | 890.0±39.74^a-c^ | 128.9±8.51^b^ | 107.7±18.89^b^ | 191.2±9.32^ab^ | 149.4±13.76^b^ |
| T5 | 298.9±7.21^a^ | 276.7±8.09^a^ | 268.7±9.80 ^a^ | 280.9±7.57 ^a^ | 583.4±25.53 ^a^ | 584.6±28.44 ^a^ | 1159.9±182.22 ^a^ | 976.5±39.26 ^a^ | 149.0±10.80 ^a^ | 174.8±2.48 ^a^ | 207.3±9.34 ^a^ | 191.6±8.18 ^a^ |
| SEm(±) | 7.09 | 10.75 | 9.05 | 8.10 | 28.29 | 43.07 | 78.50 | 46.16 | 6.47 | 10.03 | 6.55 | 11.46 |
| LSD (*P*=0.05) | 21.42 | 32.50 | 27.35 | 24.51 | 85.54 | 130.23 | 237.37 | 139.59 | 19.55 | 30.34 | 19.81 | 34.64 |

Note: SEm(±): Standard Error of Mean; LSD: Least Significant Difference; DAS: Days after sowing. T1: Control; T2: Corm dipping in 5% seaweed extract; T3: (Foliar spray of 5% seaweed extract at 30, 45, 60, 75 DAS); (Drenching with 5% seaweed extract in the soil after corm sowing at 30, 45, 60, 75 DAS); T5 (corm dipping + foliar spray with 5% seaweed extract at 30, 45, 60, 75 DAS).

Table S3. Effect of different methods of seaweed extract application on dry weight of corm and total dry weight of plant (g) of saffron at different growth intervals 45DAS and 90DAS.

| Treatment | Corm dry weight (g/plant) | | | | Total dry weight (g/plant) | | | |
| --- | --- | --- | --- | --- | --- | --- | --- | --- |
|  | 45DAS | | 90DAS | | 45DAS | | 90DAS | |
|  | 2020-21 | 2021-22 | 2020-21 | 2021-22 | 2020-21 | 2021-22 | 2020-21 | 2021-22 |
| T1 | 0.96±0.012^e^ | 0.94±0.05^de^ | 1.30±0.07^e^ | 1.47±0.11^de^ | 1.57±0.03^e^ | 1.71±0.01^c-e^ | 2.22±0.10^e^ | 2.76±0.21^de^ |
| T2 | 1.12±0.03^c^ | 1.16±0.06^b^ | 2.08±0.20^c^ | 2.37±0.14^bc^ | 2.19±0.06^b^ | 2.00±0.05^ab^ | 4.01±0.15^b^ | 3.86±0.15^a^ |
| T3 | 1.12±0.02^cd^ | 1.05±0.03^b-d^ | 2.03±0.12^cd^ | 1.78±0.11^d^ | 1.81±0.04^cd^ | 1.95±0.03^a-c^ | 3.15±0.13^cd^ | 2.86±0.10^d^ |
| T4 | 1.28±0.03^b^ | 1.15±0.03^bc^ | 2.62±0.12^b^ | 2.69±0.14^ab^ | 1.91±0.06^c^ | 1.94±0.11^a-d^ | 3.29±0.23^c^ | 3.62±0.26^a-c^ |
| T5 | 1.38±0.03^a^ | 1.32±0.05^a^ | 2.91±0.06^a^ | 2.95±0.03^a^ | 2.41±0.02^a^ | 2.17±0.10^a^ | 4.55±0.22^a^ | 3.75±0.23^ab^ |
| SEm(±) | 0.02 | 0.04 | 0.07 | 0.12 | 0.05 | 0.08 | 0.10 | 0.18 |
| LSD (*P*=0.05) | 0.07 | 0.15 | 0.22 | 0.36 | 0.15 | 0.24 | 0.32 | 0.56 |

Note: SEm(±): Standard Error of Mean; LSD: Least Significant Difference; DAS: Days after sowing. T1: Control; T2: Corm dipping in 5% seaweed extract; T3: (Foliar spray of 5% seaweed extract at 30, 45, 60, 75 DAS); (Drenching with 5% seaweed extract in the soil after corm sowing at 30, 45, 60, 75 DAS); T5 (corm dipping + foliar spray with 5% seaweed extract at 30, 45, 60, 75 DAS).

Table S4. Effect of different methods of seaweed extract application on number and weight of daughter corms of saffron.

| Treatment | Number of daughter corms/plant | | | | Total number of corms/m^2^ | | Total corm weight (g/m^2^) | |
| --- | --- | --- | --- | --- | --- | --- | --- | --- |
|  | 45DAS | | 90DAS | | At harvest | | At harvest | |
|  | 2020-21 | 2021-22 | 2020-21 | 2021-22 | 2020-21 | 2021-22 | 2020-21 | 2021-22 |
| T1 | 0.47±0.17^de^ | 0.53±0.13^c-e^ | 1.33±0.21^c-e^ | 1.53±0.29^c-e^ | 85.0±6.08^b-e^ | 68.2±4.02^b-d^ | 289.8±33.81^b-e^ | 297.5±33.62^a-c^ |
| T2 | 1.20±0.29^c^ | 0.93±0.37^c^ | 1.80±0.20^c^ | 2.07±0.39^c^ | 113.6±3.75^a^ | 68.8±3.22^bc^ | 290.1±35.81^b-d^ | 296.0±65.76^a-d^ |
| T3 | 1.07±0.19^cd^ | 0.67±0.11^cd^ | 1.66±0.28^cd^ | 1.86±0.29^cd^ | 88.8±5.65^bc^ | 67.0±1.92^b-e^ | 330.2±13.33^bc^ | 208.8±36.81^c-e^ |
| T4 | 2.00±0.37^ab^ | 1.80±0.29^ab^ | 2.60±0.22^ab^ | 3.00±0.32^ab^ | 88.0±5.98^b-d^ | 74.8±1.72^b^ | 351.4±27.55^b^ | 341.1±33.97^ab^ |
| T5 | 2.13±0.23^a^ | 2.27±0.06^a^ | 3.13±0.14^a^ | 3.66±0.06^a^ | 101.0±10.64^a^ | 88.8±2.8^a^ | 447.6±36.85^a^ | 415.1±3231^a^ |
| SEm(±) | 0.21 | 0.16 | 0.21 | 0.24 | 19.31 | 2.91 | 87.25 | 129.58 |
| LSD (*P*=0.05) | 0.64 | 0.50 | 0.64 | 0.71 | 6.38 | 8.80 | 28.85 | 42.85 |

Note: SEm(±): Standard Error of Mean; LSD: Least Significant Difference; DAS: Days after sowing. T1: Control; T2: Corm dipping in 5% seaweed extract; T3: (Foliar spray of 5% seaweed extract at 30, 45, 60, 75 DAS); (Drenching with 5% seaweed extract in the soil after corm sowing at 30, 45, 60, 75 DAS); T5 (corm dipping + foliar spray with 5% seaweed extract at 30, 45, 60, 75 DAS)

Table S5. Effect of different methods of seaweed extract application on chlorophyll and photosynthesis.

| Treatments | Chlorophyll a  (mg/g FW) | Chlorophyll b  (mg/g FW) | Carotenoid  (mg/g FW) | Photosynthesis  (μ mol/m^2^/s) |
| --- | --- | --- | --- | --- |
| T1 | 1.85±0.05^e^ | 1.37±0.05^e^ | 0.12±0.003^b-e^ | 16.3±0.30^de^ |
| T2 | 2.09±0.04^d^ | 1.55±0.06^d^ | 0.12±0.004^b-d^ | 16.7±0.44^d^ |
| T3 | 2.23±0.06^bc^ | 1.78±0.03^bc^ | 0.12±0.002^bc^ | 18.6±0.64^c^ |
| T4 | 2.30±0.04^b^ | 1.89±0.03^b^ | 0.13±0.001^b^ | 20.4±0.29^b^ |
| T5 | 2.45±0.03^a^ | 2.09±0.03^a^ | 0.14±0.003^a^ | 21.9±0.23^a^ |
| SEm(±) | 0.044 | 0.045 | 0.003 | 0.434 |
| LSD (*P*=0.05) | 0.132 | 0.136 | 0.008 | 1.311 |

Note: SEm(±): Standard Error of Mean; LSD: Least Significant Difference. T1: Control; T2: Corm dipping in 5% seaweed extract; T3: (Foliar spray of 5% seaweed extract at 30, 45, 60, 75 DAS); (Drenching with 5% seaweed extract in the soil after corm sowing at 30, 45, 60, 75 DAS); T5 (corm dipping + foliar spray with 5% seaweed extract at 30, 45, 60, 75 DAS).

Table S6: Effect of application method of seaweed extract on nutrient composition of saffron corms

| Treatment | N (mg/g) | P (mg/g) | K (mg/g) | Zn (mg/g) | Fe (mg/g) | Mn (mg/g) | Cu (mg/g) | Mg (mg/g) | Ca (mg/g) |
| --- | --- | --- | --- | --- | --- | --- | --- | --- | --- |
| T1 | 25.59±0.16^a^ | 2.30±0.08^a^ | 11.35±0.06^a^ | 0.20±0.003^a^ | 1.51±0.01^a^ | 0.03±0.005^ab^ | 0.06±0.002^a^ | 2.5±0.04^a^ | 2.8±0.02^c^ |
| T2 | 22.63±0.34^c^ | 1.25±0.06^d^ | 10.56±0.04^c^ | 0.18±0.006^b^ | 0.45±0.01^e^ | 0.02±0.004^bc^ | 0.03±0.002^b^ | 2.2±0.03^e^ | 3.1±0.02^a^ |
| T3 | 23.81±0.31^b^ | 1.84±0.12^b^ | 10.88±0.04^b^ | 0.17±0.003^b-d^ | 0.68±0.02^cd^ | 0.02±0.004^bc^ | 0.02±0.00^cd^ | 2.4±0.01^ab^ | 2.7±0.04^e^ |
| T4 | 20.44±0.33^d^ | 1.68±0.05^bc^ | 10.46±0.026^cd^ | 0.18±0.003^bc^ | 0.79±0.02^b^ | 0.04±0.00^a^ | 0.03±0.002^bc^ | 2.4±0.02^b-d^ | 2.9±0.01^b^ |
| T5 | 19.90±0.37^de^ | 1.18±0.07^de^ | 10.27±0.10^e^ | 0.18±0.003^bc^ | 0.69±0.01^c^ | 0.02±0.004^cd^ | 0.03±0.002^bc^ | 2.4±0.01^bc^ | 2.8±0.02^cd^ |
| SEm(±) | 0.22 | 0.06 | 0.06 | 0.004 | 0.015 | 0.004 | 0.002 | 0.02 | 0.02 |
| LSD (*P*=0.05) | 0.68 | 0.19 | 0.18 | 0.012 | 0.044 | 0.012 | 0.007 | 0.07 | 0.07 |
